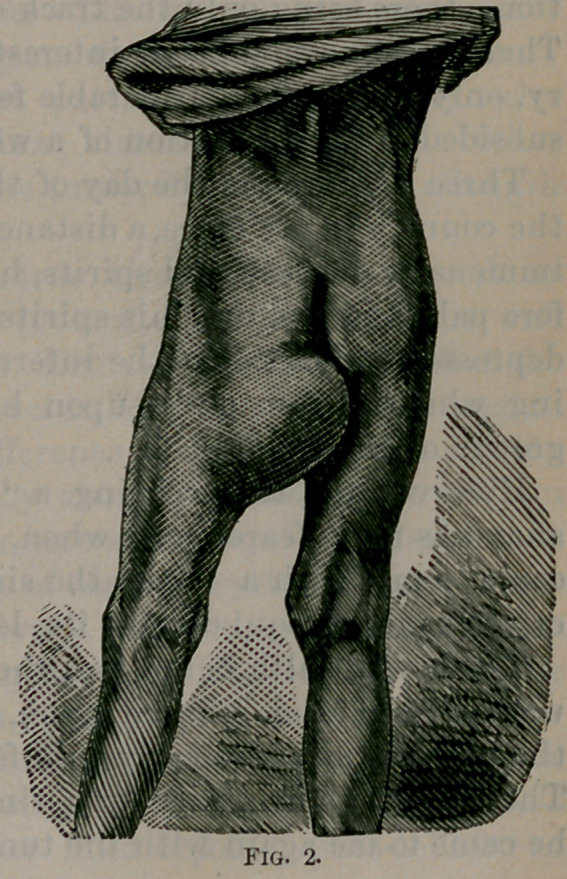# Cases in Surgery

**Published:** 1884-08

**Authors:** T. M. McIntosh

**Affiliations:** Thomasville, Ga.


### CASES IN SURGERY.

BY T. M. McINTOSH, M. D., THOMASVILLE, GA.

(*Concluded from page* 273.)

### CASE 2D, RECURRING FIBROID OF THIGH—OPERATION, RECOVERY, RECURRENCE, DEATH.

Willie B., col., age 6 years, in summer of 1879 was brought to me with a tumor on the internal border of the thigh, xtending from gluteal crease posteriorly, to the flexure of the thigh at groin, anteriorly, and downward for more than half the length of thigh, occupying over half its circumference and projecting toward and against the opposite limb to such an extent that the leg was semi-flexed, with the toe only touching the ground when standing or walking. When walking it was necessary to rotate the entire body in a kind of twisting motion, when a step was taken with either leg. This obviated to a great extent the pressure and friction incident to locomotion, but notwithstanding exercise caused an almost constant pain in the tumor.

The feel of the tumor was hard and fibrous, with a somewhat lobulated surface. The history was that three years before an enlargement was noticed on the internal surface of the thigh, about its upper third, and believed to have been caused by a blow received sometime before in that region. There had been no pain in it before it grew large enough to interfere with walking and be irritated thereby. The boy's general condition was not good, being pale, anæmic and thin. There was an intermittency of the pulse about every fifth or sixth beat, which was probably a functional disturbance, as I could detect no organic lesion.

The accompanying cuts give a very correct idea as to the appearance of the tumor at the time of operation.

On July 4th, 1879, assisted by a number of physicians, the operation for removal of the tumor was done. To render the operation bloodless, the limb was first tightly bandaged with an ordinary roller bandage, from the foot to a point above the tumor, (the femoral artery at the same time being compressed to prevent access of blood into the limb or tumor), and around the thigh at the upper margin of the bandage was made two turns of a stout rubber tube, and traction made sufficient to compress the femoral artery; by

FIG. 1.

this simple procedure deriving most of the advantages of a complete and expensive Esmarch bandage. The roller was then removed from around the tumor only, and an elliptical incision, about two inches wide, was made over the internal and most prominent surface of the tumor, beginning at a point near the ischio pubic articulation and extending obliquely downward and backward to the lowest border of the tumor on the posterior portion of the thigh. The removal was accomplished with as little cutting as possible—preferably tearing with handle of the scalpel and fingers when it could be done. A portion of the sartoris muscle was necessarily removed where it crossed the tumor and was intimately interwoven with its tissue. A firm and the most fibrous part of the tumor, was cut off from its attachment to the periosteum of the descending ramus of the pubic bone. The operation was almost entirely bloodless, not two ounces being lost. A few small arteries had to be tied.

After the completion of the operation, and some minutes after the cessation of the administration of the chloroform, just upon removal of the bandage from the limb, which had been left on during the operation, there was a prompt and alarming fall of the pulse and general marks of prostration, which continued for perhaps fifteen minutes, and slowly passed away under the

FIG. 2.

frequent administration of hypodermics of whisky and complete inversion of the body. This condition was believed to have been due to cerebral anæmia, caused by the obstruction of an amount of blood from the general circulation sufficient to fill the blood-vessels of the thigh, which had been exsanguinated by the bandage. Certainly the anæsthetic did not produce it. This to do justice to chloroform, which should only bear burdens of censure which are justly due.

The wound was dressed by placing in it a small rubber drainage tube long enough to project beyond each end of the incision an inch, drawing the lips of the wound together by stitches over this and gently compressing all by adhesive plaster strips. With a Davidson's syringe, the wound was then washed with a 1–40 solution of carbolic acid, passed through the projecting ends of the drainage tube, and over this was placed a rather thick layer of common carded cotton saturated with the same strength solution of acid. Over all this, and around the entire limb, extending some distance above and below the edges of the wound, a very thick layer of dry, carded cotton, securing all by a carefully applied roller bandage.

This dressing was not disturbed for one week, when upon removal the wound was found to have almost entirely healed by first intention—there being only the track of the drainage tube left ununited. There were no notable or interesting general features in the history, only there was considerable fever for a few days, but which had subsided at the expiration of a week.

Three weeks from the day of the operation, the boy walked from the country to my office, a distance of two miles. He had improved immensely in flesh and spirits, his cheeks were round and full, before pale and hollow; his spirits were buoyant and gleeful, before depressed and irritable, the intermittent pulse had gone—all showing what a severe drain upon his system had been the pain and growth of the tumor.

However, the tumor being a "Recurring Fibroid," its reappearance was to be feared, and when, in the following October, the boy came to me with a tumor the size of a hen's egg growing in the cicatrix about equi distant its length, it was no disappointment.

The boy's health being fine, the immediate removal of the tumor was advised and insisted upon, but the mother not appreciating the importance of an early interference, the advice was not heeded. The boy left the vicinity for a time. The latter part of December he came to me again with the tumor much larger than before the operation, with an ulceration at its apex which bled frightfully and frequently. The condition of the patient was so bad; the character of the growth so evidently malignant; its extent so vast, an operation now was not to be taken into consideration. The ulceration rapidly progressed; the bleeding continued profuse and the boy died on 1st January following from exhaustion by hemorrhage, having tetanoid convulsions the day previous to death.

I regret very much not obtaining permission to operate on the recurring tumor when seen the first time, as it offered the only hope of a permanent relief, and an almost sure prolongation of life.

Many cases are on record of such tumors being removed five, six or seven times and in some permanent immunity resulted, though unfortunately there is often a recurrence in a more malignant type than the original neoplasm.

### CASE 3D, LIPOMA OF THIGH—WEIGHT, SEVEN POUNDS.

Lela S., white, age 15. When five years of age the first appearance of the enlargement was noticed, and no cause was assigned for its appearance. I first saw the case four years ago, when the tumor was only half its present size. I then advised an operation, but the parents of the child did not desire it, as there had been no inconvenience whatever from its presence. The following measurement taken by Drs. Taylor and Withington show the size of the tumor: Circumference around the largest part, 25½ inches with muscles tense; 25 inches with muscles lax; circumference of the other thigh, 17 inches, making that of the tumor 8½ inches, with a length of about 12 inches.

I had never fully diagnosed the true character of the growth, as there was a rather unusual feature present which obscured the well marked signs of a lipomatous new formation. This was that the relaxation or contraction of the rectus femoris and tensor vagina femoris would make a marked difference in the consistence of the tumor and also its size, as shown by above measurements. When these muscles were lax the tumor bore a soft, semi-solid feel, much like a fibro-cellular, or some forms of osseous cysto-sarcoma; when tense, the feel was hard and fibrous. Under no conditions was there the lobulated character of an lipoma. Exploration with the hypodermic needle gave at times blood; again nothing. Its slow growth, freedom from pain and innocuousness to the constitution of the patient pointed to a diagnosis of benignancy. Its semi-fluid condition made one suspect either fluid lipoma, fibro-cellular or some variety of cystic tumor. The tumor had been seen a year ago by an eminent Georgia surgeon without forming a diagnosis.

While the patient was being anæsthetized the entire limb was bound tightly with a roller bandage, from the toes to the groin, and encircled above the tumor with two turns of stout rubber tubing, precisely as was done in the operation on Case 2d.

An incision twelve inches long on the external surface of the thigh was made, and the tumor removed, partly by tearing, when possible; but as it had no defined incapsulating membranes, and had grown extensively in the intermuscular spaces, and intimately incorporated much muscular tissue with its substance, considerable cutting was required. The rectus femoris having been completely surrounded by it, and incorporated within it to some extent, it was necessary to remove part of it with the tumor, also a considerable part of the tensor vaginæ femoris. Their relations to these muscles explained the influence of their contraction upon the size and consistence of the tumor.

The dressing was identical with the previous case, except a silk drainage was used instead of the rubber tubing, as my supply of the latter was exhausted.

To secure better the drainage an incision an inch long was made through the cellular tissue and skin in the lowest part of the pocket left by the removal of the tumor.

In a week's time the dressing was removed, when it was found there had been no drainage at all, the silk not subserving this purpose, though the external parts had completely united, confining within the wound its exudations, which gave marked fluctuation.

All fever and constitutional disturbance having gone on the third day, and the wound looking perfectly healthy, I suppose this fluid might have been left to be slowly absorbed, but I opened the wound at its lowest portion and let out a pint of sero-sanguinolent fluid perfectly free from odor and putrefaction. The girl, two weeks from the operation, is well. In this case I desire to call attention to the care exercised for the control of hemorrhage, and which, I believe, in Cases 1 and 2 enabled them to go on to recovery. To those who live in remote communities and who are called upon to perform operations at long distances from their offices, where there are but few conveniences and no available or intelligent assistance, this method of controlling hemorrhage with the common roller bandage and rubber tubing—a simple and convenient means of applying the principle of Esmarch's bloodless operation—will be found exceedingly efficient, useful and satisfactory.

Every physician can get it, and it costs nothing. I have used it in quite a number of various kinds of operations on the extremities, nor have I found that it ever produced any strangling of wounds or tendency thereto, after operations, as has been charged to Esmarch's bandage; and I have found it always to control the bleeding exceedingly well, which is of value to the patient, and enables one to operate much more rapidly and with less assistance, as the wound is not obscured by blood.

Also, I have been much gratified at the favorable progress and healing of all wounds dressed after the manner described in the two preceding cases. The carded cotton one can get at any farm house at no expense at all, as against a considerable cost of a little bit of a roll of absorbent cotton as found in our drug stores; and as far as the quality of the two materials is concerned as a dressing, I believe the raw material to be almost or quite as good (if applied properly) as the prepared material for the first layer of the dressing, and preferable for the external layers, as it excludes air better, preserves a more equable temperature of the wound, maintains its elasticity longer, and therefore a more equable and continuous gentle pressure. If a wound once exposed to the atmosphere be thoroughly cleansed of all blood clots and foreign material with a 1-40 carbolic acid solution, or, as I am rather inclined to believe, with warm water alone, have perfect drainage, the coöptation of the flaps firm, by a gentle and elastic pressure, the parts entirely at rest, the local temperature uniform, I do not think it necessary or desirable to dress but few wounds before a week from the time of the first dressing, and in some cases much longer.

I have been treating wounds by this plan for some time, and have had quite a number of cases upon which to test it, and my results have been better and the healing process quicker than when I made daily dressings, and therefore I think that permanent dressings and dry wounds are better, in most instances, than frequent ones and ablutions.

By a careful and painstaking use a few simple and available materials, one may get an antiseptic, if not a "Lister" dressing, and as Mr. Lister himself does not now insist upon the use of the spray and elaborate details of his method, one, I think, may feel that they have done full justice to their patient if they dress wounds in such manner as will secure all the above named conditions. Certainly all micro-organisms are not excluded, nor does their absolute exclusion prevent suppuration, or the development of constitutional symptoms, consequently we must look to conditions within our patients that influence the process of healing of wounds, which no present achievement of science has discovered, or can avert.

The permanent dressing is an old method and its value recognized, but as it is usually made with "Lister'' details, its benefits have been confined to the extensively equipped hospital or city surgeon, and those who are not so well supplied, believing that they have not at their command the means by which to obtain the most rapid wound repair, do not devote that care to their surgical dressings which they would if they thought good would come of it.

Every one has the needed materials, I think, and if they will only operate and dress their wounds in the manner described in the last two cases they will be much rewarded.

Progress is not alone in the direction of the discovery of new means and new methods, but it is also made in the enlightened and diligent use of agents we already possess.

## Figures and Tables

**Fig. 1. f1:**
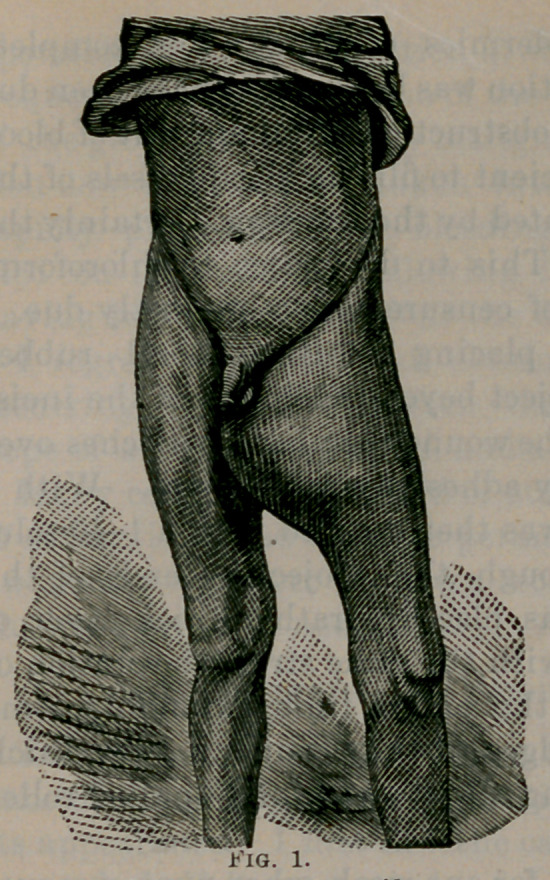


**Fig. 2. f2:**